# Leukemia Prediction Using Sparse Logistic Regression

**DOI:** 10.1371/journal.pone.0072932

**Published:** 2013-08-30

**Authors:** Tapio Manninen, Heikki Huttunen, Pekka Ruusuvuori, Matti Nykter

**Affiliations:** 1 Department of Signal Processing, Tampere University of Technology, Tampere, Finland; 2 Institute of Biomedical Technology, University of Tampere, Tampere, Finland; Cardiff University, United Kingdom

## Abstract

We describe a supervised prediction method for diagnosis of acute myeloid leukemia (AML) from patient samples based on flow cytometry measurements. We use a data driven approach with machine learning methods to train a computational model that takes in flow cytometry measurements from a single patient and gives a confidence score of the patient being AML-positive. Our solution is based on an 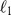 regularized logistic regression model that aggregates AML test statistics calculated from individual test tubes with different cell populations and fluorescent markers. The model construction is entirely data driven and no prior biological knowledge is used. The described solution scored a 100% classification accuracy in the DREAM6/FlowCAP2 Molecular Classification of Acute Myeloid Leukaemia Challenge against a golden standard consisting of 20 AML-positive and 160 healthy patients. Here we perform a more extensive validation of the prediction model performance and further improve and simplify our original method showing that statistically equal results can be obtained by using simple average marker intensities as features in the logistic regression model. In addition to the logistic regression based model, we also present other classification models and compare their performance quantitatively. The key benefit in our prediction method compared to other solutions with similar performance is that our model only uses a small fraction of the flow cytometry measurements making our solution highly economical.

## Introduction

Leukemias are a common malignancy of blood cells emerging from different cell types [1]. For example, acute myeloid leukemia (AML), which is the focus of this work, emerges during myeloid differentiation. However, the traditional classification of leukemias relies predominantly on morphologic and cytochemical features of the tumor cells rather than the developmental origin of the malignancy [2].

Blood cancers are diagnosed with various techniques including features from morphologic, cytochemic, cytogenetic, and flow cytometry. While morphologic, cytochemic, and cytogenetic analysis include standard pathological stainings that lead to low dimensional data that can typically be interpreted directly under microscope, analysis of flow cytometric data includes interpretation of more complex high dimensional data distributions and, thus, computer assisted decision systems are needed to support diagnosis decision making. [3].

Flow cytometry can be used to analyze a large number of individual cells and, thus, is well suited to detect, e.g., cells that express particular cancer related surface markers from blood samples. In the measurement process, cells are labeled with fluorescent dye. The dye can be delivered, e.g., by specific antigen binding antibodies. Fluorescent labeled cells are guided through a laser beam and the resulting fluorescence and scatter parameters, typically forward and side scatter, are detected by photo detector. Forward scatter is informative of the cell size and side scatter corresponds to cell granularity. These control measures are used to make sure the data from different experiments are comparable by “gating” or selecting the cells with corresponding features for subsequent analysis. With modern instruments several lasers and dyes with different wave lengths can be used and, thus, multiple measurements can be done from a single cell in parallel. [3], [4].

In this paper, we introduce a supervised prediction method for diagnosis of AML from flow cytometry data. Our prediction model is data driven in the sense that no prior biological knowledge is used in the model construction, e.g., in feature selection. There are two stages in the processing: First, for each individual flow cytometry tube with a different combination of measured fluorescence markers, a feature generation and dimension reduction steps are applied after which a probability density model is learned from the training data for both AML-positive and healthy patients. Second, error scores calculated between the test patient's distribution and the learned distributions from each tube are aggregated by a logistic regression model that predicts the final confidence score of the patient being AML-positive. The parameters of the logistic regression model are estimated from the training data using 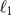 regularization, which works as an embedded feature selector and results in a sparse model where only a part of the tubes are needed in making the diagnosis. The feature selection is our key contribution: Our prediction model requires significantly fewer measurements than the alternative approaches.

The prediction model presented in this paper is based on our submission to the DREAM6/FlowCAP2 Molecular Classification of Acute Myeloid Leukaemia Challenge (DREAM6 AML Challenge) held in conjunction with the Dialogue for Reverse Engineering Assessments and Methods (DREAM6) conference organized in 2011. In the challenge evaluation, 8 of the 17 participating teams, including our solution, achieved 100% prediction result in terms of ranking based error metrics (e.g., precision and recall) when tested against the hidden test data consisting of measurements from 20 AML-positive and 160 healthy patients. Other well performing models were based, e.g., on *Kullback-Leibler* divergence based distance between histogram estimated densities (http://www.ehu.es/biologiacomputacional/team21_vilar) and *Learning Vector Quantization* (LVQ) with moment based features by Biehl et al. [5]. Further description about the challenge results and joint algorithm descriptions from the participating teams is published in [6].

The fact that about half of the participating teams achieved a perfect result suggests that the challenge test data was not particularly difficult to classify. However, the *training data* contains a couple of “difficult patients” that were misclassified by many teams. This works as a motivation for this paper, where we combine the challenge training and test sets and their golden standards to get a larger set of 43 AML-positive and 316 healthy patients. Moreover, due to the high accuracy of several methods, we will concentrate on finding the *simplest model* that is able to reach a comparable performance. The classifiers are compared using cross-validation (CV) with area (AUC) under the operating characteristics (ROC) curve and the precision-recall (PR) curve as well as confusion matrices.

The performance of our predictor is compared against the methods by Vilar and by Biehl et al. [5]. Both solutions achieved a 100% prediction accuracy in the DREAM6 AML Challenge. Further, Vilar had the highest correlation between the predictor output and the ground truth. Notice that the Pearson correlation used in the challenge evaluation is not a very good evaluation metric (although probably the only applicable one in the challenge) because it depends, e.g., on the link function of the prediction model, which is irrelevant from the classification point of view.

Further in this paper, an improved prediction method is proposed that simplifies our original method without decreasing its performance. In addition, we apply a simple linear discriminant analysis (LDA) on the average intensities of each fluorescent marker and use it as a baseline reference method. Finally, we use all the data for training and analyze the created model revealing useful information from the viewpoint of medical AML diagnosis. Especially the coefficients of the sparse logistic regression model are inspected in order to find out, which of the flow cytometry tubes and markers are actually needed in the diagnosis and which can be discarded.

## Materials and Methods

The following sections give a description of the flow cytometry data that we use in the experiments. In addition, we give a brief overview and a block diagram of the method that we use to automatically diagnose AML followed by a detailed algorithm description. Finally, we introduce the other models, which we use for comparison in the experiments.

### Flow Cytometry Data

We use the DREAM6 AML prediction challenge data set which is available at the challenge website (http://www.the-dream-project.org/challenges). The data set consists of flow cytometry measurements taken from 43 AML-positive and 316 AML-negative patients. The original challenge training set is a subset of these consisting of 179 patients (23 AML-positive and 156 healthy ones).

Flow cytometry is used for quantification of expression of different protein markers in the patient's blood or bone marrow sample. A limited number of protein markers can be measured for cells in one sample tube (aliquot). The challenge data contains seven tubes containing 6764 to 49370 events, i.e., cells. A different combination of five different markers is measured from each tube. These marker combinations (FL1–FL5) have been listed in Table 1. Each marker is only present in one of the tubes except for marker CD45, which is included in all of the tubes. In addition to fluorescence intensities, forward and side scatter readings are provided for each tube. Tube number eight is a control tube with non-specific-binding antibodies. We don't use this tube for prediction purposes.

**Table 1:** Fluorescence markers provided in DREAM6 AML prediction challenge data.

	FL1	FL2	FL3	FL4	FL5
Tube 1	IgG1-FITC	IgG1-PE	CD45-ECD	IgG1-PC5	IgG1-PC7
Tube 2	Kappa-FIT	Lambda-PE	CD45-ECD	CD19-PC5	CD20-PC7
Tube 3	CD7-FITC	CD4-PE	CD45-ECD	CD8-PC5	CD2-PC7
Tube 4	CD15-FITC	CD13-PE	CD45-ECD	CD16-PC5	CD56-PC7
Tube 5	CD14-FITC	CD11c-PE	CD45-ECD	CD64-PC5	CD33-PC7
Tube 6	HLA-DR-FITC	CD117-PE	CD45-ECD	CD34-PC5	CD38-PC7
Tube 7	CD5-FITC	CD19-PE	CD45-ECD	CD3-PC5	CD10-PC7
Tube 8	N/A	N/A	N/A	N/A	N/A

Seven tubes (and one unspecified control tube) with different fluorescence markers (FL1–FL5) are provided in the DREAM6 AML prediction challenge data set. (Table from challenge web site: http://www.the-dream-project.org/challenges/dream6flowcap2-molecular-classification- acute-myeloid-leukaemia-challenge).

Both raw and preprocessed flow cytometry data are provided in the challenge website. As the original experimental setup has been designed by the the contest organizers for a large benchmark study [6], we rely that the setup is meaningful and that the provided data has been processed according to the standards of the field. For prediction, we use the preprocessed data, which is compensated/translated using the method described in [7]. Briefly, the preprocessed data contains the forward scatter in linear scale, side scatter in logarithmic scale, and the fluorescence intensities for the five channels described in Table 1 in logarithmic scale. As an alternative to the preprocessed data, we experimented with a regression based preprocessing method [8] directly applied for the raw data. However, no improvement was gained in the results. Thus, all results presented below are for the preprocessed contest data which is available in CSV format from the contest web site.

### Prediction Model Overview

The flow cytometry data we are using consists of seven tubes with thousands of cells each. All the cells originate from a single patient who is either AML-positive or healthy. Instead of trying to classify each individual cell as AML-positive or healthy, the key strategy is to consider the cell populations as whole and, finally, fuse together the information that each tube independently provides.

**Figure 1:**
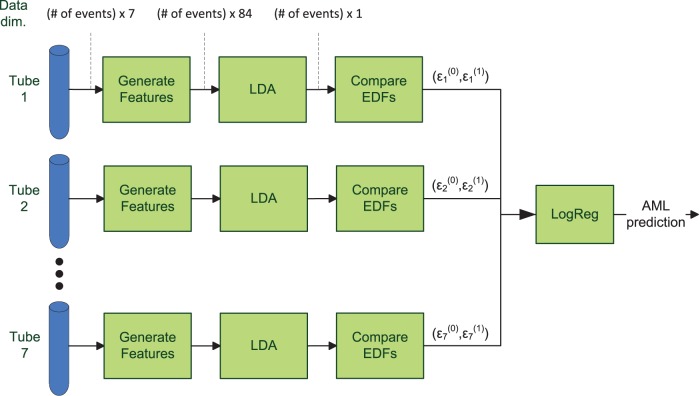
Block diagram of the AML predictor. First, for each tube, more features are generated from the seven dimensional flow cytometry data by calculating all multiplications and divisions of any two basic features. Second, the data is mapped into 1-d with LDA and the EDF of the resulting data is compared with the corresponding training EDFs of both AML-positive and healthy populations. Finally, the 14 EDF similarity scores are combined by a logistic regression model that outputs a confidence value of the test patient being AML-positive. Similar to the reference EDFs, LDA coefficients maximizing the separation between AML-positive and healthy populations as well as logistic regression coefficients are estimated in a training phase.

A detailed description of the structure of our prediction model will be given in the forthcoming sections. For an overview, see Figure 1. As a starting point, we have a feature generation step where the dimensionality of the tube data is increased by generating some artificial features. This is done in order to avoid the limitations of the subsequent linear model, thus, improving the class separation. Next, the resulting 84-dimensional data is projected to 1-d by using Fisher's linear discriminant analysis (LDA) that maximizes the separability between the AML-positive samples and the healthy ones. We then compare the empirical cumulative distribution function (EDF) of the tube data with corresponding trained EDFs of both AML-positive and healthy populations by using a simple mean squared error metric. This results in two EDF similarity measures per tube.

For the higher level aggregation of individual tubes we use logistic regression. The parameters of the logistic regression model are estimated using a method that penalizes the coefficient magnitudes using an 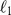 penalty [9]. This results in a sparse model where only a few tubes are needed in evaluating the predictor output. An implementation of our predictor is available as MATLAB source code from http://www.cs.tut.fi/~hehu/DREAM6-AML.html.

### Feature Generation

Our prediction model uses rather simple linear operations and distribution comparison in only one dimension. Thus, we cannot expect the model to discover very complicated relations between the input features. For this reason, we artificially expand the set of features from the initial 5 fluorescence intensities and 2 scatter features by taking all possible inverses (7 pcs) and second powers (7 pcs) of single features and multiplications (21 pcs) and divisions (42 pcs) of any combination of two features. Thus, the dimensionality is increased from 7 to 84.

While overlearning seems not to be a problem in increasing the dimensionality of the data, there are, however, some practical problems. Namely, the average storage requirement of an 84-dimensional data matrix of a single tube is between 4 and 32 Mb depending on the number of events in the tube. When training with all our data, this gets multiplied by 359, i.e., the number of patients. This means that we have over 11 Gb of training data in one of the tubes, which can be a problem depending not only on the hardware setup but also on the type of forthcoming processing.

### EDF Comparison

For each tube with a particular population of cells the next step is to determine whether that population originates from an AML-positive or healthy patient. This is done by using a two-sample distribution comparison test such that we compare the estimated distribution of the tested tube data against the distributions of both AML-positive and healthy tubes, which have been estimated from the training data. As a result, we get two similarity measures.

Multidimensional (in our case, 84-dimensional) density estimation is known to be a difficult problem and the situation is further complicated by the large amount of data. Our solution is to first use LDA to map each sample 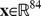 into 1-d:
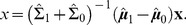
(1)


Here, 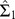 and 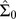 are the sample covariance matrices and 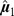 and 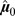 are the sample means of both AML-positive and healthy training samples, respectively.

Applying the LDA significantly reduces the amount of data and allows us to use traditional 1-d methods for distribution comparison. LDA allows us to use training data for finding the linear mapping that best separates the AML-positive and healthy populations in 1-d. Further, we do not need to have 11 Gb of free memory in our computer, because we can easily calculate the required mean vectors and covariance matrices iteratively one patient at a time.

For 1-d two-sample distribution comparison, we calculate two similarity measures 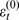 and 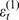 for each of the tubes 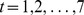. The measures are based on the mean squared error (MSE) between the EDFs of the tested tube data and the one that has been trained for either healthy or AML-positive tubes, respectively:
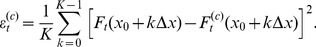
(2)


In the above equation, 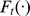 is the EDF of the tested tube data after LDA mapping, and 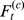 are the corresponding EDFs of the trained healthy tube (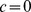) and the trained AML-positive tube (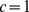).

Instead of averaging the squared error in Equation 2 over the complete EDFs, the error is only evaluated in 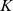 discrete points uniformly chosen between points 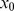 and 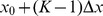. Our validation tests show that we can use value as low as 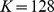 without degrading the classification performance. Thus, the training EDFs are parametrized with 128 parameters, which frees us from carrying the whole training data with us in the testing phase. We choose 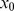 equal to the minimum of the training samples and 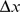 such that 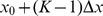 equals to the maximum of the training samples.

In addition to the EDF MSE in Equation 2, also more established distribution comparison methods were tested including *two-sample Cramér-von Mises test* [10], *Kolmogorov-Smirnov test* [11], and *Kullback-Leibler divergence* [12]. Further, error measures like correlation and MSE between the probability density functions estimated with kernel smoothing density estimation were tested. However, based on validation results, EDF MSE gave the best classification performance.

### Sparse Logistic Regression

Given a 14-dimensional feature vector 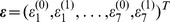 corresponding to the EDF error measures of each tube as defined in Equation 2, our strategy is to use the logistic regression model
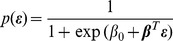
(3)to estimate the probability 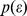 of the patient being AML-positive. Parameters 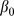 and 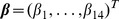 are estimated from the training data by maximizing the 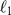-penalized log-likelihood
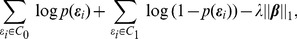
(4)where 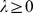 is the regularization parameter and 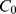 and 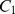 are the training sets of healthy and AML-positive patients, respectively.

Similar to that in linear regression using *Least Absolute Shrinkage and Selection Operator (LASSO)* [9], the 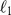 penalty in Equation 4 results in the solution vector 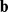 being sparse such that only a few of the coefficients are non-zero. Thus, the penalization works as an implicit feature selector and any tube irrelevant from the diagnosis point of view gets automatically dropped out from the model.

The role of the parameter 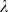 in Equation 4 is to control the amount of regularization: the larger the value of 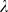, the heavier the regularization. For small values of 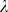, the solution is close to the maximum likelihood solution, while large values of 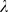 allow restricted solutions only and push the coefficients towards zero. In our case he value of 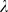 is automatically determined by cross-validation.

Regularized generalized linear models including logistic regression models can be efficiently fit using a coordinate descent algorithm proposed by Friedman et al. [13]. There is also a MATLAB implementation available at http://www-stat.stanford.edu/~tibs/glmnet-matlab.

### Reference Methods

In the forthcoming experiments section, we compare our prediction model with the method by Vilar (http://www.ehu.es/biologiacomputacional/team21_vilar) and the method by Biehl et al. [5] (http://www.the-dream-project.org/story/code). In addition to these, we test two simple approaches as a reference: an 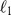 regularized logistic regression classifier and an LDA classifier with plain mean values of the marker intensities (or scatter values) as features.

The basic idea in Vilar's predictor is similar to our method. First, the distributions of data from AML-positive and healthy training patients are compared against that of the tested patient for each tube. Second, the resulting distribution similarity scores are aggregated in a logistic regression model to derive an AML confidence score between 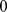 and 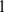.

Vilar's method doesn't apply explicit dimension reduction for the data to alleviate the problem of multidimensional density estimation. Instead, the probability density of the 7-dimensional tube data is approximated by using multiple lower dimensional densities. These densities are constructed such that only one of the fluorescence markers FL1, FL2, FL4, or FL5 is taken into account in each density estimate while fluorescence marker FL3 (which is always CD45 regardless of the tube) and forward and side scatters are present in all of the low dimensional densities. This results in 4 different 4-dimensional distributions instead of one 7-dimensional. In practice, these densities are approximated by histograms with 9 bins per dimension uniformly spaced in logarithmic scale between 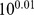 and 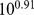 for all but the side scatter for which the corresponding range is between 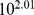 and 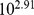. An important feature, found out by our validation tests, is that any sample outside the range of the histogram is considered an outlier and discarded.

The comparison of the distributions in Vilars's method is done by using Kullback-Leibler divergence, i.e., *relative entropy*. Finally, the entropies from each tube are combined by simply summing together the relative entropies with the AML-positive population and subtracting the entropies with healthy population. This total entropy is then mapped with the logistic function to get the final AML confidence score. In terms of the notation in Equation 3 this means that 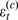 equals to the relative entropy against class 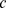 in tube 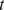 and that the model coefficients are not estimated from training data but fixed to 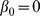 and 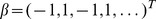.

Biehl's method differs from our and Vilar's by not trying to estimate the full densities of the data. Instead, six quantities, mean, standard deviation, skewness, kurtosis, median, and interquantile range, are calculated for each marker and used as features. For classification, Biehl uses *Generalized Matrix Relevance Learning Vector Quantization* (GMLVQ). See [5] for details.

## Results

This section presents the experimental results. First, we run a CV test to assess and compare the performance of the previously described five different methods: our original, Vilar's, and Biehl's methods, and 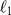 regularized logistic regression and LDA with plain mean values of the marker intensities as features. In the result tables and figures, these five methods are referred as *EDF-MSE/LR-LASSO*, *Vilar*, *Biehl et al.*, *Mean/LR-LASSO*, and *Mean/LDA*, respectively. Second, we use all the available data to train the two sparse logistic regression predictors and analyze the resulting models.

### Performance Assessment

We use the combined DREAM6 AML Challenge training and test data consisting of 43 AML-positive and 316 healthy patients and stratified 10-fold CV to benchmark the prediction models. Thus, the validation result estimates the generalized model performance when using about 90% of the data, i.e., 39 AML-positive and 284 healthy patients, for training.

**Figure 2:**
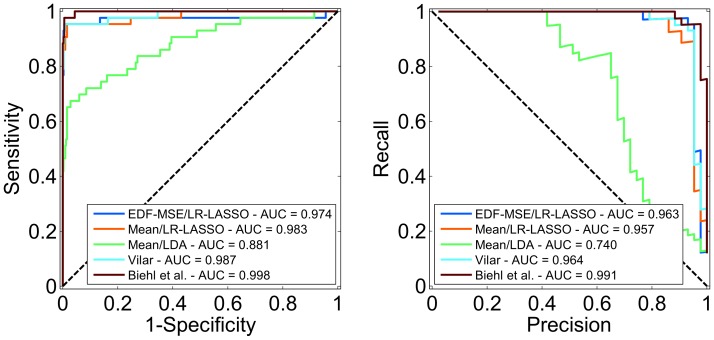
ROC and PR curves of 10 times repeated 10-fold CV test. The left panel shows the ROC and the right one the PR curves. The legend shows the AUC values for different predictors. Except for LDA (green line), the differences in AUC values are statistically insignificant.

The ROC and PR curves resulting from the 10-fold CV are shown in Figure 2 together with the AUC values for each predictor. According to the AUC analysis tool StAR [14], only LDA with mean features (AUC = 0.88) differs from the other methods (AUC 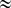 0.98) with statistical significance. The StAR tool is based on a two-sided Mann-Whitney test with a null hypothesis that two AUC values are the same. A confidence level of 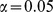 was used in the significance test. Exact p-values are given in Table 2.

**Table 2:** P-values of the significance test testing the difference of the AUC ROC values between each pair of prediction methods.

Method	Biehl et al.	Vilar	Mean/LR-LASSO	EDF-MSE/LR-LASSO	Mean/LDA
Biehl et al.	–	–	–	–	–
Vilar	0.1598	–	–	–	–
Mean/LR-LASSO	0.1537	0.6809	–	–	–
EDF-MSE/LR-LASSO	0.2509	0.3486	0.6509	–	–
Mean/LDA	0.0003	0.0004	0.0019	0.0040	–

The null hypothesis is that the AUC ROC values between two methods are the same. Given p-values are provided by the online ROC analysis tool StAR [14].

**Figure 3:**
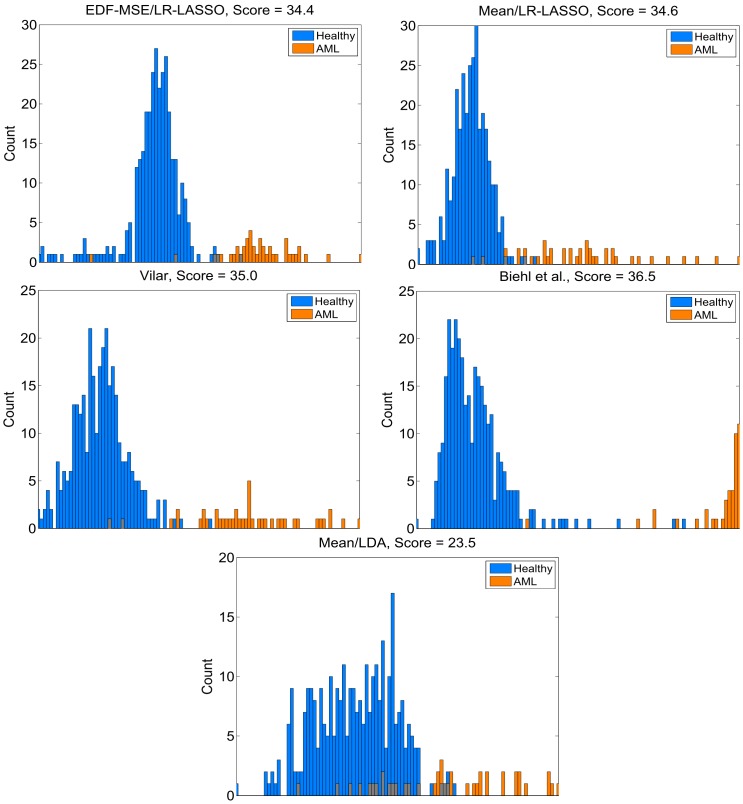
Distribution of the test samples in predictor output space. Colors of the histogram bars indicate the true class label (gray color denotes the overlapping parts). Titles in each plot tell the corresponding prediction method and a score value calculated as the negative log10 p-value that the class separation has been attained by chance.

Figure 3 shows how the test samples of the CV procedure distribute into the predictors' output space shown on the 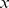-axis. For visualization purposes, the output of Biehl's method has been rescaled by taking the power of 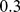. The 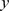-axis shows the histogram count. The title in each plot shows the corresponding prediction method and a score value, which has been calculated as a p-value that the AUCs of the ROC and PR curves have been achieved by random chance. The p-value is given in negative log-10 scale, i.e., the higher the better. See [15] for more details on how the score has been derived.

**Figure 4:**
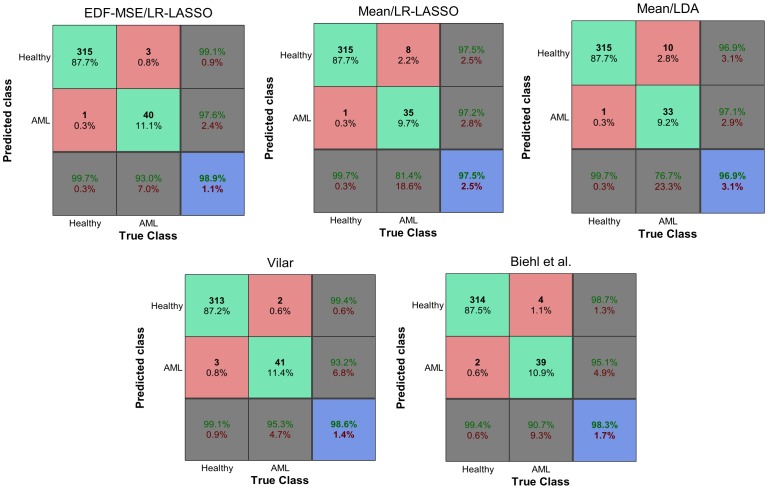
Confusion matrices. Distribution of the 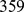 test patients into false/true positives/negatives in a 10-fold CV test. Summary statistics are given in the last rows/columns.

Figure 4 shows the confusion matrices after thresholding the predictor output. For logistic regression models, the threshold is 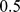. Biehl et al. don't give a specific threshold for the output of the GMLVQ classifier. In this case, we have chosen the threshold such that the classification error in the training data is minimized.

### Final Model

In this section, we use all the available 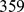 patients for training the logistic regression classifier with 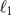 regularization. In the case of the EDF MSE features the trained classifier can be written as
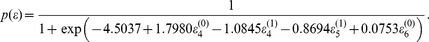
(5)


The remarkable notion is that the 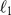 regularization only selects 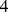 of the available 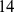 features. Further, only tubes 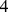, 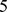, and 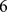 are included in the final model making tubes 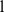, 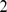, 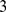, and 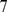 non informative from the diagnosis point of view.

Based on the absolute values of the logistic regression coefficients in Equation 5, tube number 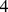 (see corresponding markers from table 1) seems to be the most important one. Also tube 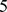 has a substantial effect on the predictor output but only when comparing against AML-positive (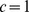) patients. Tube number 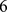 has a similar effect on the healthy patients. However, relatively small absolute coefficient value makes tube 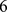
's contribution rather insignificant. Notice that comparing absolute coefficient values makes sense because the similarity scores 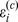 are normalized.

**Figure 5:**
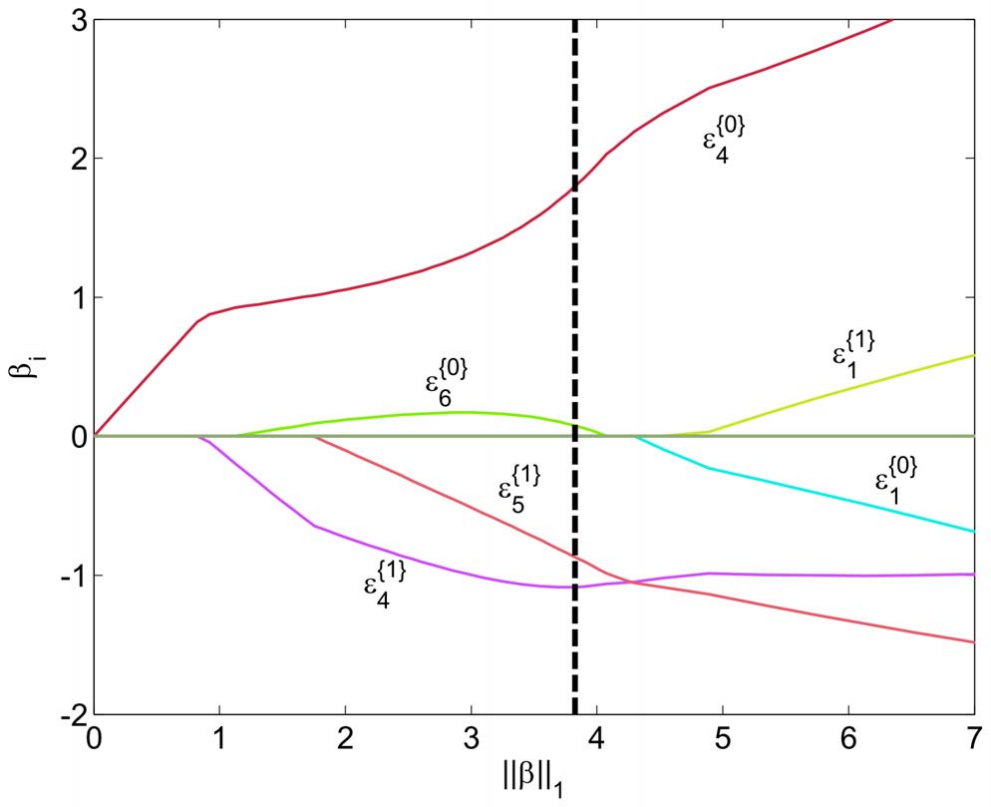
Regularization path of the 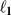 regularized logistic regression model with EDF MSE features. Coefficient values have been plotted with respect to the 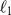 norm of the coefficient vector. The black dashed line shows the final model chosen by cross-validation.

The full regularization path of the above case is shown in Figure 5. The final model indicated by the black dashed line is chosen by minimizing 10-fold CV error. The EDF distance features from tube 4 are the first ones taken into the model when the regularization is gradually decreased (i.e., 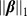 increases). This further emphasizes the importance of tube 4. Interestingly, 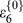 is removed from the model right after the point of minimum CV error suggesting that similar performance could be attained by using only tubes 4 and 5. Notice, how the features from tube 1 approximately differ only by their sign. Further, the signs seem to be the wrong way around: smaller distance to the healthy population contributes towards classifying the patient as AML positive and the vice versa. This is probably due to overlearning.

**Figure 6:**
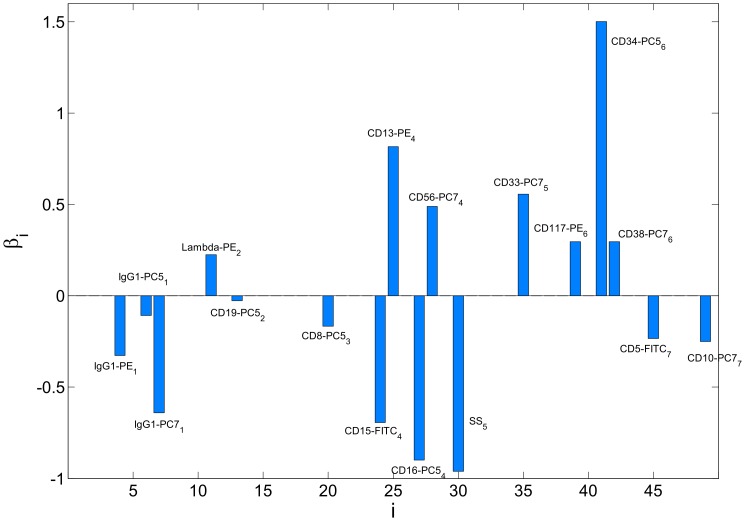
Logistic regression model coeffients with average normalized scatter/intensity values as features. Only 17 of the 49 coefficients get a non-zero value due to 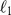 regularization. Numbers in the subscript indicate the tube number.

A similar analysis was run on the simpler model where the 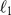 regularized logistic regression was applied on the mean values of each marker intensity or scatter reading. In this case, 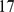 of the 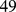 variables are automatically selected according to the CV error. Figure 6 shows the coefficient values of the logistic regression model. About half of the contribution in terms of 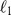 norm of the coefficient vector comes from the four most significant markers, which are CD34-PC5 from tube 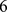, side scatter from tube 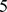, and CD16-PC5 and CD13-PE from tube 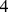.

More detailed information about the whole regularization path of the above model is shown in Table 3. Here, we have picked the best feature sets with given size along the regularization path. Set sizes span from zero (bias term only) to 17, which is the model with lowest CV error also shown in Figure 6. The markers in the list have been sorted according to the absolute value of their corresponding coefficient such that the most important feature is always on top. The tube number of each marker is shown in the subscript. Especially CD34-PC5, side scatter (SS), and CD16-PC5 seem to be included in the model from very early stage of the regularization path.

**Table 3:** Best subsets of certain size along the regularization path of the logistic regression classifier with average marker intensities as features.

Set size	0	1	2	3	4	5
Markers		CD34-PC5_6_	CD34-PC5_6_	CD34-PC5_6_	CD16-PC5_4_	SS_6_
			SS_6_	SS_6_	CD34-PC5_6_	CD16-PC5_4_
				CD15-FITC_4_	SS_6_	CD34-PC5_6_
					CD15-FITC_4_	CD38-PC7_6_
						CD56-PC7_4_
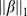	0.00	0.05	0.16	0.29	0.92	1.74
CV err.	0.120	0.120	0.120	0.120	0.092	0.053
**Set size**	**6**	**7**	**8**	**9**	**10**	**11**
**Markers**	**SS_6_**	**SS_6_**	**SS_6_**	**SS_6_**	**SS_6_**	**SS_6_**
	CD16-PC5_4_	CD16-PC5_4_	CD16-PC5_4_	CD16-PC5_4_	CD16-PC5_4_	CD16-PC5_4_
	CD34-PC5_6_	CD34-PC5_6_	CD34-PC5_6_	CD34-PC5_6_	CD34-PC5_6_	CD34-PC5_6_
	CD38-PC7_6_	CD38-PC7_6_	CD38-PC7_6_	CD38-PC7_6_	CD38-PC7_6_	CD38-PC7_6_
	CD56-PC7_4_	CD56-PC7_4_	CD56-PC7_4_	CD56-PC7_4_	CD56-PC7_4_	CD56-PC7_4_
	CD117-PE_6_	CD117-PE_6_	CD13-PE_4_	CD13-PE_4_	CD13-PE_4_	CD13-PE_4_
		CD13-PE_4_	CD117-PE_6_	CD33-PC7_6_	CD33-PC7_5_	CD33-PC7_5_
			CD33-PC7_5_	CD117-PE_6_	CD15-FITC_4_	CD15-FITC_4_
				CD15-FITC_4_	CD10-PC7_7_	CD10-PC7_7_
					CD117-PE_6_	CD117-PE_6_
						CD19-PE_7_
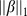	2.40	2.50	3.11	3.57	4.31	4.41
CV err.	0.033	0.033	0.025	0.022	0.019	0.019
**Set size**	**12**	**13**	**14**	**15**	**16**	**17**
**Markers**	**SS**_6_	**SS**_6_	**CD34-PC5**_6_	**CD34-PC5**_6_	**CD34-PC5**_6_	**CD34-PC5**_6_
	CD16-PC5_4_	CD34-PC5_6_	SS_6_	SS_6_	SS_6_	SS_6_
	CD34-PC5_6_	CD16-PC5_4_	CD16-PC5_4_	CD16-PC5_4_	CD16-PC5_4_	CD16-PC5_4_
	CD56-PC7_4_	CD56-PC7_4_	CD13-PE_4_	CD13-PE_4_	CD13-PE_4_	CD13-PE_4_
	CD38-PC7_6_	CD13-PE_4_	CD56-PC7_4_	CD56-PC7_4_	CD56-PC7_4_	CD15-FITC_4_
	CD13-PE_4_	CD38-PC7_6_	CD38-PC7_6_	CD38-PC7_6_	CD15-FITC_4_	IgG1-PC7_1_
	CD33-PC7_5_	CD33-PC7_5_	CD33-PC7_5_	CD33-PC7_5_	CD33-PC7_5_	CD33-PC7_5_
	CD10-PC7_7_	CD10-PC7_7_	CD15-FITC_4_	CD15-FITC_4_	IgG1-PC7_1_	CD56-PC7_4_
	CD15-FITC_4_	CD15-FITC_4_	IgG1-PC7_1_	IgG1-PC7_1_	CD38-PC7_6_	IgG1-PE_1_
	CD19-PE_7_	CD19-PE_7_	CD10-PC7_7_	CD10-PC7_7_	CD10-PC7_7_	CD38-PC7_6_
	CD117-PE_6_	CD117-PE_6_	CD19-PE_7_	CD19-PE_7_	IgG1-PE_1_	CD117-PE_6_
	CD5-FITC_7_	CD5-FITC_7_	CD117-PE_6_	CD117-PE_6_	CD117-PE_6_	CD10-PC7_7_
		IgG1-PC7_1_	CD5-FITC_7_	CD5-FITC_7_	CD5-FITC_7_	CD5-FITC_7_
			SS_5_	SS_5_	CD19-PC5_2_	Lambda-PE_2_
				IgG1-PE_1_	CD8-PC5_3_	CD8-PC5_3_
					Lambda-PE_2_	IgG1-PC5_1_
						CD19-PC5_2_
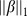	4.66	4.87	5.95	6.02	7.21	8.49
CV err.	0.019	0.019	0.019	0.017	0.014	0.011

Markers in the list have been sorted according to the absolute value of their corresponding coefficient such that the most important feature is always on top. CV err. indicates the 10-fold CV classification error when using regularization parameter 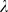 corresponding to the 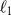 norm of the coefficient vector as given in 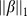. The lowest CV error (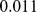) is attained with the 17 markers shown in Figure 6.

The above analysis of the most important features supports the discussion of [5], where seven markers were recognized as the key features: forward scatter, side scatter, CD15-FITC, CD117-PE, CD16-PC5, CD34-PC5, and CD10-PC7. All of these appear in our final model (Figure 6) or on the regularization path (Table 3) except the forward scatter measure. Missing forward scatter makes sense because in [5] they show that the predictive power of the forward scatter on linear scale relies on it's higher moments, especially standard deviation and skew, rather than on the distribution average, which we use in our model.

In the model with marker means as features, scatters and CD45 intensities were not combined over the different tubes but the mean values from each tube were taken as individual features. Similar to that in Biehl's method [5], also pooling of these variables was tested. However, this decreased the validation performance. To our knowledge, the intensity values of each marker should be independent of the tube they are measured from. One explanation for the above phenomenon is that there is some kind of overlearning occurring when not combining the corresponding markers from separate tubes.

## Discussion

In this paper, we have described an automated method for AML diagnosis from flow cytometry data. Our method uses the combined DREAM6 AML Challenge training and test data for which we run a cross-validation test in order to assess our predictor. In addition, we compare our predictor against predictors by Vilar and Biehl et al. [5], which were among the best performing methods in the DREAM6 AML Challenge. Experimental results show that there are no statistically significant differences between the best performing prediction methods when cross-validating them against the challenge data set of 43 AML-positive and 316 AML-negative patients. Cross-validated classification performances reach 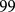 %.

The key benefit in our predictor model is that, in the training phase, the 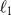 regularized logistic regression model automatically estimates the relevance of each flow cytometry tube or marker. Only a subset of the available features (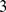 of the 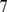 tubes with the EDF MSE features or 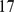 of the 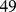 markers with the mean intensity features) are selected still reaching a performance equivalent to the alternative methods. Thus, in a testing phase, only a fraction of the usual flow cytometry measurements are needed. Another beneficial feature in our model is that it has virtually no parameters that would need manual tuning. Instead, the regularization parameter 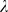 is automatically chosen by cross-validation and the number of EDF bins 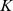 is, by construction, robust against changes in the data. This is in contrast, e.g., with Vilar's algorithm where even a small change in the dimensions of the density histogram was noticed to result in deterioration of the cross-validation performance by several percentage units.

The proposed framework contains two alternatives for feature extraction: The simple version with mean intensity features (termed Mean/LR-LASSO in the experiments) and the complex version using the mean squared errors between the EDFs (termed EDF-MSE/LR-LASSO). Experiments show that there is no significant difference in performance between the two alternatives. In fact, all the studied methods are almost perfect in terms of accuracy, so the only reasonable conclusion is to favor the simplest solution. Many studies emphasize that simplicity should be the guiding principle in the design of classifiers; see for example [16], [17]. In [17], the author describes this reasoning as follows: “…simple methods typically yield performance almost as good as more sophisticated methods, to the extent that the difference in performance may be swamped by other sources of uncertainty…”. Along these lines, we believe that the proposed use of regularized logistic regression also has the best generalization for future samples, due to its simplicity. In particular, according to the experiments of this paper, the method of choice should use simple mean intensity features (Mean/LR-LASSO) instead of the complex EDF-MSE features.

However, we do believe that the more complex EDF-MSE features has potential to simplify the classifier structure even further, allowing even better generalization performance for future samples. In the experiments, the complex feature extraction model trained with all data uses slightly more features than the simple feature extraction (21 versus 17), but when trained with the subset defined as training data in the DREAM6 challenge, the resulting model uses only two tubes, i.e., 14 features. One reason for the fluctuation in the selected features is seen in the model of Eq. (5), where only one of the two similarity measures 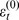 and 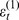 is used for tubes 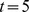 and 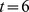. The obvious choice would be to exploit them both, which happens when trained with the training subset of DREAM6. We plan to investigate this further by generalizing the method to use Group LASSO for logistic regression [18], which allows the feature selection in groups.

In comparison to the binary diagnosis task studied in this paper, a more clinically relevant and computationally more challenging question is how to accurately predict the specific subtype of AML. For example, the World Health Organization has issued a classification of AML subtypes that aims to be more clinically relevant by containing prognostic value and provides guidelines for treatment [2]. Data driven methods could provide accurate and objective classification to existing subgroups, by solving a multi class classification problem, and class discovery methods could be used to uncover new homogeneous subtypes from data. However, to address these questions, more extensive data collections need to be generated. By using multi class logistic regression classifier, our methods could directly be extended to multi class classification problem and it would be of interest to see whether the tubes and features that were deemed uninformative in context of AML classification would in fact contain information about differences between AML subtypes and, further, which tubes give information about specific AML subtypes. This type of approach has previously provided good results, e.g., in studying human brain activity created by different types of visual stimuli [19].
